# Mature Cystic Teratoma of Liver

**Published:** 2013-05-02

**Authors:** Richa Gupta, Kalpana Bansal, Vivek Manchanda, Ruchika Gupta

**Affiliations:** Department of Pathology, Chacha Nehru Bal Chikitsalaya, Geeta Colony, Delhi - 110031, India; Department of Radiology, Chacha Nehru Bal Chikitsalaya, Geeta Colony, Delhi - 110031, India; Department of Pediatric Surgery, Chacha Nehru Bal Chikitsalaya, Geeta Colony, Delhi - 110031, India; Department of Pathology, Chacha Nehru Bal Chikitsalaya, Geeta Colony, Delhi - 110031, India

**Keywords:** Mature teratoma, Liver, Histopathology

## Abstract

A four-year-old boy presented with constipation and mild abdominal distention for one year. Radiologic investigations showed a multiloculated cystic lesion in the caudate lobe of liver with focal calcification in the wall. The child underwent laparotomy with marsupialization of the cystic lesion. Histopathologic examination showed mature teratoma of liver.

## INTRODUCTION

Teratomas are germ cell tumors occurring most commonly in ovaries or testis. Liver is an extremely rare site for teratoma, constituting less than 1% of all teratomas [1]. An extensive review of literature yielded less than 50 cases of primary hepatic teratomas [2]. Teratomas have distinctive imaging characteristics allowing an accurate pre-operative diagnosis in majority of cases. In certain instances, radiologic diagnosis may not be very apparent, and histologic examination renders the definite diagnosis along with assessment of immature component, the latter being important for further management [3]. This case is being presented for rarity and the difficulty in radiologic diagnosis in this patient.

## CASE REPORT

A 4-year boy was brought with complaints of constipation, loss of appetite, mild abdominal distension, and intermittent fever for the last one year. Physical examination revealed pallor. There was no icterus or cyanosis. On abdominal examination, mild distension was noted. Liver was palpable extending up to the right lumbar region; it was firm, non-tender with smooth surface. Spleen was not palpable. Routine investigations revealed anemia (hemoglobin 10.5g/dl). Other hematological and biochemical investigations, including liver function tests were within reference ranges. Ultrasonography (USG) of abdomen showed hepatomegaly with a multiloculated round cystic lesion in the caudate lobe. The lesion measured 4.3x4.5x4cm and showed internal septation. No calcification or hyperechoic foci were seen. Contrast-enhanced computed tomography (CECT) scan also showed a well-defined contrast enhancing multiloculated cystic lesion involving the caudate lobe of liver. The lesion measured 43.8x43x41mm and showed multiple septations and calcification (Fig. 1). Hydatid serology (IgG by ELISA) was negative. Intra-operatively a multiloculated cyst 4 cm in diameter was seen in the caudate lobe of liver. The cyst contained clear fluid with calcification in the wall. Partial excision of the lesion and marsupialization was performed. Histopathology showed mature teratoma composed of mature elements including skin, bone, glial tissue, tooth enamel and pulp, lymphoid aggregates and choroid plexus (Fig. 2). Immature component was not seen in any of the sections examined. Postoperative alpha-fetoprotein (0.82 ng/ml) and beta–human chorionic gonadotrophin (1.2 mIU/L) performed were within reference ranges. The child is doing well one year after surgery and follow-up USG did not reveal any residual mass/recurrence.

**Figure:**
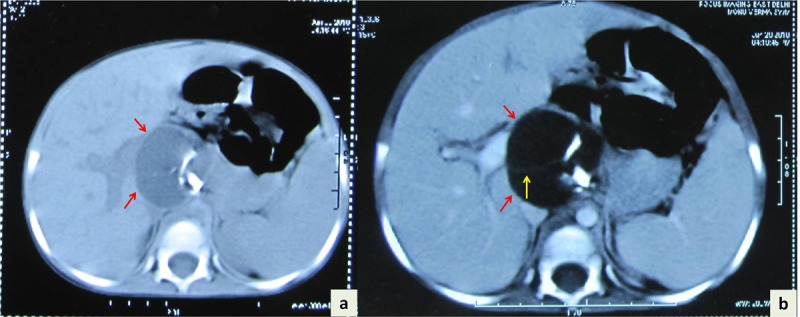
Figure 1: Axial view of computed tomography (CT) scan showing a well-defined rounded hypodense lesion (arrows) in caudate lobe of liver (a). Contrast-enhanced CT scan image of the same demonstrating multicystic lesion (red arrows) with thin septa (yellow arrow) involving caudate lobe of liver (b).

**Figure:**
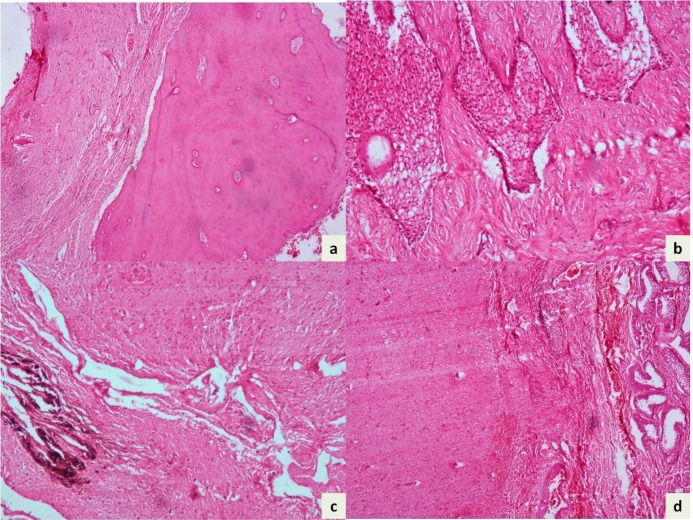
Figure 2: Photomicrographs showing glial tissue, bone (a, H and E x40), squamous epithelium (b, H and E x100), pigmented epithelium (c, H and E x40) and glands (d, H and E x40).

## DISCUSSION

Most hepatic teratomas are encountered in patients less than 3 years of age. In pediatric patients, teratomas account for less than 1% of all hepatic neoplasms [2]. Majority of these tumors are discovered incidentally due to site-specific mass effect. Tumors in liver cause abdominal distension, nausea and vomiting due to compression of adjacent structures. Hepatic teratomas have been reported more commonly in female patients and involve right lobe of liver in many cases [2]. Our patient was a boy and had the lesion in caudate lobe of liver. 

Ultrasonography (USG) of teratoma shows hypo- or anechoic component representing cystic portion and hyperechoic foci denoting calcifications or macroscopic fat. Fat-fluid levels, due to presence of sebum, are considered pathognomonic of teratoma [4]. On CT scan, calcifications are seen as hyperattenuating and macroscopic fat as hypoattenuating foci within a cystic cavity in a teratoma. CT scan also permits characterization of fluid as sebum, serous or complex [5]. Occasionally, there may be absence of calcification, making the radiologic diagnosis difficult [7]. A minority of teratomas do not demonstrate sebum-filled cystic cavity [7]. Few cases of mature teratoma have none to minimal fat on radiologic studies [7]. In our patient also, USG and CT scan did not show fat in the wall of the cystic cavity or sebum in it. The presence of a cystic lesion in the liver with calcification in the wall of the cyst resembled the radiologic findings of a non-calcified hydatid cyst, hence this impression was considered in our differentials. 

A definitive diagnosis of mature teratoma is possible by histologic examination alone. Histologically, teratomas contain variable amounts of bone, hair, skin, sebum, fat, muscle, neural tissue and rarely pancreatic and thyroidal tissue. Teratomas can be categorized as benign or malignant on the basis of their histopathological features [2]. Hence, surgical resection is the mainstay of treatment, since the presence of immature tissue of any germ layer adversely affects the prognosis [3]. In our case, no immature component was found and the patient has been doing well with no residual mass. However, the risk of recurrence looms large since the child underwent marsupialization of the cyst. Hence, regular follow-up at three or six month intervals, including ultrasound examination, is being performed.

## Footnotes

**Source of Support:** Nil

**Conflict of Interest:** None declared

## References

[1] Certo M, Franca M, Gomes M, Machado R (2008). Liver teratoma.. Acta Gastroenterol Belg.

[2] Rahmat K, Vijayananthan A, Abdullah Bjj, Amin Sm (2006). Benign teratoma of the liver: a rare cause of cholangitis.. Biomed Imaging Interv J.

[3] Souftas V, Polychrondis A, Giatromanolaki A, Perente S, Simopoulos C (2008). Dermoid cyst in the hepatoduodenal ligament: report of a case.. Surg Today.

[4] Pakdirat B, Prachaphinyo T, Pakdirat P (1994). Radiology of retroperitoneal cystic teratoma in adult: a case report.. J Med Assoc Thai.

[5] Davidson A J, Hartman D S, Goldman S M (1989). Mature teratoma of the retroperitoneum: radiologic, pathologic, and clinical correlation.. Radiology.

[6] Ayyappan A P, Singh S E, Shah A (2007). Mature cystic teratoma in the falciform ligament of the liver.. J Postgrad Med.

[7] Occhipinti K A, Frankel S D, Hricak H (1993). The ovary: computed tomography and magnetic resonance imaging.. Radiol Clin North Am.

